# The American Practitioner: A Monthly Journal of Medicine and Surgery

**Published:** 1871-01

**Authors:** 


### Book Notices.

The American Practioner: A Monthly Journal of Medicine and Surgery. Edited by David W. Yandell, M.D., and Theophalus Parvin, M.D. Published by J. P. Morton & Co., Louisville, Ky.

We have received from the publishers, the first and second volumes of this journal, neatly bound in cloth. Each volume contains six monthly numbers of the *Practitioner*, and together embrace the issues for 1870. It is one of the best and most practical medical periodicals in this country.

